# Direct visualization endoscopic retrograde appendicitis therapy versus laparoscopic appendectomy for management of acute uncomplicated appendicitis

**DOI:** 10.1055/a-2638-6177

**Published:** 2025-08-07

**Authors:** Jun-yu Pan, Hui-xin Zhi, Jie-li Chen, Hao-xin Chen, De-feng Li, Jun Yao, Li-sheng Wang

**Affiliations:** 1 Department of Gastroenterology, Shenzhen People’s Hospital, The Second Clinical Medical College, Jinan University, Shenzhen, 518020, China

**Keywords:** Endoscopy Lower GI Tract, Stenting, Pancreatobiliary (ERCP/PTCD), Cholangioscopy

## Abstract

**Background and study aims:**

Direct visualization endoscopic retrograde appendicitis therapy (ERAT), an advanced technique building upon conventional ERAT, represents a novel endoscopic approach for managing acute uncomplicated appendicitis. This study aimed to assess clinical efficacy and safety of employing cholangioscope-guided endoscopic intervention as a therapeutic approach.

**Patients and methods:**

A retrospective analysis was conducted on 656 patients presenting with suspected acute appendicitis between February 2024 and November 2024. To minimize baseline differences, propensity score matching was applied, resulting in a final study population of 34 patients undergoing direct visualization ERAT and 68 patients treated with laparoscopic appendectomy (LA). Key outcome measures included technical and clinical success rates, operative time, time to postoperative pain resolution, length of hospital stay, recurrence rate, incidence of adverse events (AEs), and overall patient satisfaction.

**Results:**

The technical success rate was 97.06% (33/34) in the ERAT group and 100% in the LA group ( *P* = 0.333), while clinical success was achieved in 94.12% (32/34) of ERAT cases compared with 100% in the LA cohort ( *P* = 0.109). Notably, ERAT was associated with a significantly shorter operative time (37 vs 50 minutes; *P* < 0.001) and more rapid postoperative pain relief ( *P* = 0.001), with a greater proportion of patients reporting complete symptom resolution within 2 days of the procedure. There were no significant differences between the two groups in terms of AEs, length of hospital stay, or patient satisfaction. During follow-up, a recurrence of appendicitis was observed in one ERAT patient (2.94%, 1/34).

**Conclusions:**

Direct visualization ERAT demonstrated high feasibility and effectiveness as a diagnostic and therapeutic modality for acute uncomplicated appendicitis, offering a promising alternative to conventional approaches.

## Introduction

The appendix is a narrow, blind-ended tubular structure situated at the junction of the cecum and ileum. Historically regarded as a vestigial organ, recent research has unveiled its important role in immune function, as it harbors abundant lymphoid tissue and contributes to regulation of gut microbiota 1 2 3 4 . Consequently, preserving the appendix during treatment of appendiceal diseases may hold significant clinical value. Acute appendicitis is the most prevalent cause of acute abdominal conditions, with a reported incidence ranging from 6.7% to 8.6%, and its prevalence continues to rise 5 . Although surgical intervention remains the standard treatment, the issue of negative appendectomy, a scenario in which up to 25% of resected appendices are later found to be histologically normal, poses a considerable challenge 3 6 7 .

Endoscopic retrograde appendicitis therapy (ERAT) has emerged as a novel, minimally invasive approach for both diagnosis and treatment of acute appendicitis 8 . First introduced by Liu, ERAT applies principles akin to those of endoscopic retrograde cholangiopancreatography (ERCP), a technique widely utilized for managing biliary obstructions 9 10 . Compared with surgery, ERAT not only offers rapid symptom relief but also preserves the appendix, a distinct advantage. Originally designed for biliary tract visualization, the single-operator cholangioscope (SOC; Micro-Tech Co., Ltd., Nanjing, China) has been successfully adapted for managing acute uncomplicated appendicitis. As a state-of-the-art direct visualization instrument, SOC is increasingly recognized as a viable and innovative alternative in clinical practice 11 12 .

Despite its potential, clinical application of cholangioscope-guided ERAT for acute appendicitis remains inadequately explored, largely due to lack of direct comparative studies with conventional appendectomy. This study aimed to systematically assess clinical efficacy, safety, and limitations of cholangioscope-guided direct visualization ERAT in treatment of acute appendicitis. Herein, we present our findings to provide insights into the utility of this innovative technology.

## Patients and methods

### Study design and participants

This retrospective clinical study analyzed data collected between February 2024 and November 2024 from the Second Clinical Medicine College (Shenzhen People's Hospital) of Jinan University, Guangdong, China. All enrolled patients were hospitalized.

Eligible participants were those diagnosed with acute uncomplicated appendicitis confirmed by abdominal computed tomography (CT) or ultrasonography, presenting with clinical symptoms and physical signs consistent with acute appendicitis, a symptom duration of less than 1 month, and who opted for either direct visualization ERAT or laparoscopic appendectomy (LA). Exclusion criteria encompassed patients with evidence of peri-appendiceal abscess, appendiceal perforation, or appendiceal gangrene on imaging; suspected chronic appendicitis with intermittent symptoms; colorectal malignancy; known allergies to anesthetics; inability to undergo colonoscopy or surgery due to poor general health; or missing follow-up data.

### Procedure

Direct visualization endoscopic retrograde appendicitis therapy for treatment of acute uncomplicated appendicitis.Video 1

**Fig. 1:**
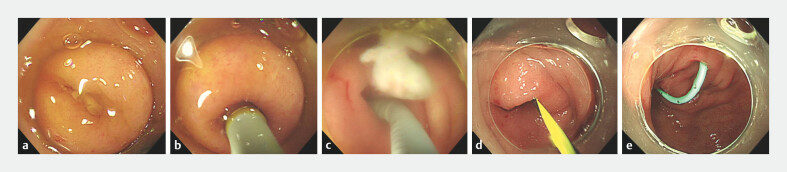
Procedures for direct visualization using cholangioscope. **a** Identification of the appendiceal orifice. **b** Intubation with the cholangioscope. **c** Thorough irrigation of the appendiceal lumen. **d** Intubation with the yellow Zebra guidewire. **e** Placement of the appendiceal stent.

For ERAT procedures, patients fasted for at least 6 hours before undergoing a colonoscopy. Bowel preparation consisted of oral administration of 2000 mL of polyethylene glycol electrolyte solution 4 to 6 hours prior to the procedure, followed by dimethicone 10 minutes before anesthesia to minimize foam in the intestinal lumen, with the Boston Bowel Preparation Scale ensuring a minimum score of 7. Procedures were performed by physicians with over 10 years of experience in gastrointestinal endoscopy and expertise in ERCP techniques, using a colonoscope (CF-HQ290I, Olympus, Tokyo, Japan, 3.7-mm channel diameter), a cholangioscope (CDS22001, Micro-tech, Nanjing, China, channel diameter > 1.0 mm, working length 2200 ± 50 mm), a Zebra guidewire (Boston Scientific, United States), and a single-pigtail pancreatic stent (5F, 5 cm, Cook, Ireland). Following bowel preparation, a transparent cap was attached to the colonoscope distal end. Under general anesthesia, the colonoscope was advanced to the terminal ileum for examination of the mucosa surrounding the ileocecal valve and appendiceal orifice ( Fig. 1 **a** ).

**Fig. 2:**
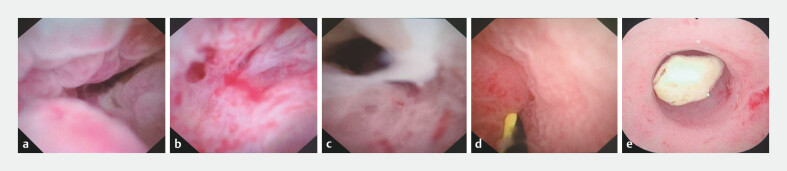
Findings observed under direct visualization using cholangioscope. **a** Swollen inner wall. **b** Mucosal congestion and bleeding. **c** Accumulation of purulent discharge. **d** Lumen narrowing. **e** Adherence of appendiceal fecalith.

Direct visualization ERAT for diagnosing and treating acute appendicitis was performed under direct endoscopic visualization and involved multiple steps, all of which were conducted without use of fluoroscopic guidance ( Video 1 ). First, Gerlach’s valve was displaced using a transparent cap to improve visualization of the appendiceal opening. If no obstructions or suspicious tumors were detected, the cholangioscope was inserted through the colonoscope channel and carefully advanced along the appendix lumen ( Fig. 1 **b** ). The appendix lumen was then examined under direct visualization, focusing on the mucosa and contents such as fecaliths or secretions, with photographs taken up to the appendix base ( Fig. 2 ). For synchronized treatment, the approach varied based on the findings. In cases of swollen appendicitis, the lumen was irrigated repeatedly with saline or metronidazole while applying negative pressure to aspirate pus until clear ( Fig. 1 **c** ). Small fecaliths were typically flushed out with repeated irrigation, but for larger obstructions, additional methods such as controlled inflation, continuous flushing, or using an extraction basket might be necessary. If a significant amount of pus or lumen narrowing was observed, a yellow Zebra guidewire was inserted into the appendix to guide placement of a 5F, 5-cm single-pigtail pancreatic stent, ensuring adequate drainage ( Fig. 1 **d,e** ). After confirming successful drainage, the cholangioscope was withdrawn slowly, followed by the colonoscope. For patients whose appendiceal obstruction could not be resolved, elective retreatment or conversion to surgical intervention was considered. In the LA group, pneumoperitoneum was established using a 10-mm supraumbilical trocar to provide laparoscopic access. A 5-mm primary port was placed in the left inguinal region, and a 5-mm secondary port was positioned subumbilically. The base of the appendix was doubly ligated with absorbable sutures, followed by resection of the distal portion. Hemostasis was achieved using electrocautery prior to specimen retrieval through the secondary port. The procedure was completed only after a final laparoscopic inspection confirmed absence of active bleeding.

All patients were hospitalized, and sedatives or analgesics were administered as needed to manage intolerable pain prior to treatment. Postoperatively, antibiotics were provided until body temperature and inflammatory markers returned to normal. Discharge decisions were based on clinical evaluations, including resolution of symptoms and absence of abdominal signs such as tenderness or rebound tenderness. In the ERAT group, patients who showed clinical deterioration after the procedure were promptly referred for surgical intervention.

### Measurements

Data were collected for all participants, including demographic variables (gender and age), body mass index, and comorbidities, expressed as frequencies and percentages. Inflammatory markers, including white blood cell count and C-reactive protein (CRP) were recorded before the procedure. Pain levels were assessed using a standardized visual analog scale (VAS), where 0 represented no pain, and 10 represented the worst pain; a score of 0 indicated complete relief of abdominal pain. Post-discharge follow-up data were collected via phone, including information on clinical outcomes, postoperative complications, and overall patient satisfaction, which was rated on a three-point scale: excellent, moderate, or terrible.

### Outcomes

Technical success in the ERAT group was defined as successful cholangioscope intubation and completion of thorough irrigation, as specified in Fig. 1 . In the LA group, it referred to complete excision of the appendix without intraoperative complications. Clinical success was defined as complete resolution of symptoms without recurrence of appendicitis. Recurrence was characterized by reappearance of significant right lower abdominal pain during the follow-up period. Adverse events (AEs) included postoperative wound hemorrhage, diarrhea, abdominal distension, nausea or vomiting, fever, and disease progression. Primary outcomes of the study were technical success rate and clinical success rate. Secondary outcomes included operative time, time to postoperative pain relief, length of hospital stay, recurrence rate, incidence of AEs, and patient satisfaction.

### Statistical analysis

Statistical analyses were performed using IBM SPSS version 26.0 and R version 4.5.0. To minimize intergroup differences, propensity score matching (PSM) was employed. Propensity scores were estimated using multivariable logistic regression models adjusted for clinically relevant covariates. A 1:2 nearest-neighbor matching algorithm was applied with a caliper width of 0.2 standard deviations and without replacement. Categorical variables were reported as frequencies and percentages. Continuous variables were presented as medians with interquartile ranges (IQRs). Categorical variables were compared using the Chi-square test or Fisher’s exact test, while continuous variables were analyzed using the Mann–Whitney U test. *P* < 0.05 was considered statistically significant.

## Results

### Patient demographics

**Fig. 3:**
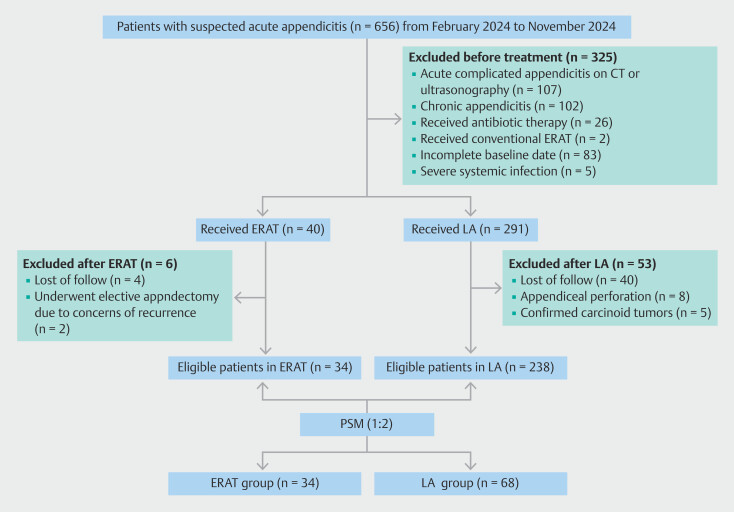
Flow diagram of patient selection.

Between February and November 2024, a total of 656 patients hospitalized for suspected acute appendicitis at our institution were screened for eligibility. Based on predefined exclusion criteria ( Fig. 3 ), 384 patients were excluded. Pretreatment exclusions (n = 325) included 102 cases of chronic appendicitis, 107 cases of complicated appendicitis found on preoperative examination (49 perforations, 40 gangrenous, and 18 periappendiceal abscesses), two patients who underwent conventional ERAT, five patients with severe systemic infections, 26 patients treated with antibiotics alone, and 83 cases with incomplete baseline data. Post-treatment exclusions (n = 59) consisted of four ERAT patients lost to follow-up, two ERAT patients who underwent elective appendectomy due to concerns of recurrence, and eight LA patients excluded for appendiceal perforation, five LA patients pathologically confirmed carcinoid tumors, and 40 LA cases lost to follow-up. After applying PSM, the final study cohort comprised 34 patients treated with direct visualization ERAT and 68 who underwent LA. Apart from duration of symptoms prior to admission, there were no statistically significant differences in baseline characteristics between the two groups. These characteristics are detailed in Table 1 .

**Table:** **Table 1** Baseline characteristics of study patients.

	Before PSM			After PSM		
	**ERAT** (n = 34)	**LA** (n = 238)	*P*	**ERAT** (n = 34)	**LA** (n = 68)	*P*
Male, n (%)	15 (44.12)	106 (44.54)	0.963	15 (44.12)	26 (38.24)	0.568
Age, years; median (IQR)	37.5 (32–44)	37 (27–46)	0.418	37.5 (32–44)	38.5 (27–47.5)	0.739
BMI, kg/m ^2^ ; median (IQR)	22.93 (20–23.7)	22.75 (20.68–24.65)	0.443	22.93 (20–23.7)	22.18 (19.61–24.7)	0.673
Body temperature ≥ 37.3°C, n (%)	1 (2.94)	27 (11.34)	0.223	1 (2.94)	3 (4.41)	1.000
WBC > 10×10 ^9^ /L, n (%)	9 (26.47)	151 (63.45)	**<** 0.001	9 (26.47)	27 (39.71)	0.187
Neutrophil ratio > 75%, n (%)	12 (35.29)	156 (65.55)	**<** 0.001	12 (35.29)	29 (42.65)	0.475
Appendicolith on CT or ultrasonography, n (%)	7 (20.59)	61 (25.63)	0.525	7 (20.59)	17 (25.00)	0.620
CRP > 5 mg/L, n (%)	24 (70.59)	155 (65.13)	0.530	24 (70.59)	55 (80.88)	0.241
VAS score; median (IQR)	3 (2–5)	4 (3–6)	0.005	3 (2–5)	4 (3–4)	0.397
Duration of symptoms prior to admission, days; median (IQR)	3.5 (2–7)	1 (1–2)	**<** 0.001	3.5 (2–7)	2 (1–3)	0.005 ^*^
Comorbidities, n (%) ^†^	4 (11.76)	19 (7.98)	0.506	4 (11.76)	7 (10.29)	1.000
*The duration of symptoms prior to admission with significant difference after PSM. †Comorbidities include diabetes, hypertension and heart disease. BMI, body mass index; CRP, C-reactive protein; CT, computed tomography; ERAT, endoscopic retrograde appendicitis therapy; IQR, interquartile range; LA, laparoscopic appendectomy; PSM, propensity score matching; VAS, visual analog scale; WBC, white blood cell.						

### Technical characteristics of direct visualization ERAT

In the ERAT group, all patients tolerated bowel preparation well, with no reported exacerbation of abdominal pain or need for modifications to the preparation protocol. Among the 34 patients, 33 (97.06%) successfully underwent cholangioscope intubation. In one patient, the procedure was discontinued due to severe luminal stenosis, which impeded cholangioscope advancement. Given the elevated risk of appendiceal perforation, neither guidewire insertion nor stent placement was attempted. This patient underwent laparoscopic appendectomy 2 days later, confirming gangrenous appendicitis. Stents were successfully placed to drain the lumen in 25 patients; however, in one case, repeated guidewire migration prevented stent placement. All eight patients with fecaliths underwent flushing to facilitate removal. A summary of procedurel characteristics for the ERAT group shown in Table 2 .

**Table:** **Table 2** Procedures during direct visualization ERAT.

Successful cholangioscope entry (n = 33)*	No. of patients (%)
Procedures	
Thorough irrigation	33 (100%)
Fecalith removal	8 (24.24%)
Stent placement	25 (75.76%)
Intraoperative cholangioscope findings	
Swollen inner wall	25 (75.76%)
Narrowing of the lumen	7 (21.21%)
Mucosal congestion and bleeding	2 (6.06%)
Accumulated purulent discharge	24 (72.73%)
Fecal fecaliths adherence	8 (24.24%)
Intraoperative appendiceal perforation	0
*In one patient (1/34), cholangioscope insertion was unsuccessful due to severe lumen stenosis.	

### Comparison of operative and post-procedure results

Among the two groups, ERAT failed in one patient. The technical success rate was 97.06% (33/34) in the ERAT group and 100% (68/68) in the LA group ( *P* = 0.333), with no statistically significant difference between the two groups. Operative time for ERAT (37 minutes, IQR 33–44 minutes) was significantly shorter than that for LA (50 minutes, IQR 42–74 minutes) ( *P* < 0.001). Analysis of pain relief duration revealed a significantly different distribution between the two groups ( *P* = 0.001), with a higher proportion of ERAT patients achieving adequate pain relief within 2 days postoperatively. No significant differences in AEs or length of hospital stay were observed between groups. Operative and post-procedure outcomes for both groups are summarized in Table 3 .

**Table:** **Table 3** Operative and post-procedure outcomes of direct visualization ERAT vs LA.

	**ERAT** (n = 34)	**LA** (n = 68)	*P*
Technical success, n (%)	33 (97.06) ^*^	68 (100.00)	0.333
Clinical success, n (%)	32 (94.12) ^†^	68 (100.00)	0.109
Recurrence of appendicitis, n (%)	1 (2.94)	0	0.333
Operative time, minutes; median (IQR)	37 (33–44)	50 (42–74)	< 0.001
Length of hospital stay, days; median (IQR)	4 (3–6)	4 (3–5)	0.728
Time to postoperative			0.001
pain resolution, n (%)			
0–1 days	24 (70.59)	25 (36.76)	
1–2 days	9 (26.47)	28 (41.18)	
> 2 days	1 (2.94)	15 (22.06)	
Adverse events, n (%)	3 (8.82)	8 (11.76)	0.748
Disease progression, n (%)	1 (2.94) ^*^	0	0.333
Post-intervention fever, n (%)	0	3 (4.41)	0.549
Diarrhea (> 3 episodes/day), n (%)	0	1 (1.47)	1.000
Abdominal distension (>24 hours), n (%)	1 (2.94)	0	0.333
Vomiting (≥ 2 episodes/day), n (%)	1 (2.94)	2 (2.94)	1.000
Wound bleeding, n (%)	0	2 (2.94)	0.551
^*^ One patient was unable to undergo cholangioscope insertion and developed gangrenous appendicitis. ^†^ One patient failed to undergo cholangioscope intubation and one experienced recurrent appendicitis. ERAT, endoscopic retrograde appendicitis therapy; IQR, interquartile range; LA, laparoscopic appendectomy.			

### Post-treatment follow-up

Both groups of patients were followed for a median duration of 5 months (IQR: 4–7). During this period, one patient (2.94%, 1/34) in the ERAT group experienced recurrence of appendicitis, occurring at 3 months post-procedure. This patient opted for LA and achieved complete symptom resolution. Including the patient in whom cholangioscope intubation failed, the clinical success rate in the ERAT group was 94.12% (32/34). In contrast, no cases of recurrent appendicitis occurred in the LA group, resulting in a 100% clinical success rate (68/68). There was no statistically significant difference in clinical success rates between the ERAT and LA groups ( *P* = 0.109). Regarding satisfaction, a higher proportion of ERAT patients (88.24%, 30/34) expressed willingness to recommend the treatment to others, compared with 79.41% (54/68) in the LA group; however, this difference was not statistically significant ( *P* = 0.520). Overall patient satisfaction is summarized in Table 4 .

**Table:** **Table 4** Comparison of patient satisfaction between direct visualization ERAT and LA.

	**ERAT** (n = 34)	**LA** (n = 68)	*P*
			0.520
Excellent, n (%)	30 (88.24)	54 (79.41)	
Moderate, n (%)	3 (8.82)	11 (16.18)	
Terrible, n (%)	1 (2.94)	3 (4.41)	
ERAT, endoscopic retrograde appendicitis therapy; IQR, interquartile range; LA, laparoscopic appendectomy.			

## Discussion

The etiology of appendicitis remains uncertain, with the most widely accepted hypothesis pointing to appendiceal blockage followed by bacterial invasion. Historically, appendectomy has been considered the "gold standard" treatment for appendicitis 13 14 . However, surgery poses risks, including negative appendectomy and postoperative complications, and has been linked to increased likelihood of developing Crohn's disease 15 16 . As a minimally invasive, nonsurgical alternative, ERAT has emerged as a safe and effective approach for managing appendicitis since its introduction. Drawing parallels with treatment of biliary tract diseases, SOC has been widely adopted for visualized interventions, suggesting potential for similar efficacy in appendicitis management. Kong et al. first reported cholangioscope-assisted ERAT for appendicitis in a small cohort study, and its effectiveness was subsequently corroborated in a retrospective survey by Tao 12 17 . Cholangioscope-assisted ERAT offers real-time, direct visualization of the appendiceal lumen, enabling precise identification and targeted treatment of underlying pathology. Furthermore, this technique eliminates need for x-ray guidance, thereby avoiding radiation exposure for both patients and endoscopic operators, making it a safer alternative to conventional ERAT methods 18 .

In this retrospective study, we employed PSM to compare direct visualization ERAT with LA in treatment of acute uncomplicated appendicitis. The results demonstrated no significant differences between the two groups in terms of technical and clinical success rates, patient satisfaction, AEs, or recurrence. Notably, 97.06% of patients (33/34) in the ERAT group achieved complete pain relief within 2 days of treatment, a significantly higher rate than that observed in the LA group (77.94%, 53/68). This finding highlighted the ability of direct visualization ERAT to effectively and rapidly alleviate pain symptoms. In addition, the ERAT group had significantly shorter operative time, thereby improving operator workflow efficiency.

According to a meta-analysis, approximately 6.01% of patients treated with conventional ERAT experience recurrence during the follow-up period 19 . In comparison, our study, conducted with a median follow-up duration of 5 months, observed recurrence in only one patient. This lower recurrence rate could be attributed to enhanced procedure precision offered by direct visualization, which likely ensures more comprehensive removal of pus and fecaliths, key contributors to recurrence risk. Alternatively, the relatively shorter follow-up period in our study might have led to underestimation of the true recurrence rate, because longer follow-up durations tend to capture additional recurrent cases, providing a more reliable assessment of long-term outcomes.

Notably, use of drainage stents remains a topic of debate, with no established consensus guiding their placement. The decision to place a drainage stent often depends on operator clinical judgment. Although some researchers advocate for adequate drainage as an effective strategy to minimize recurrence risk, our study found that drainage stents were placed in the one patient who experienced recurrence. This observation might reflect the limitations of our relatively small sample size. Further investigations are warranted to clarify factors influencing appendicitis recurrence. Post-discharge, we do not routinely recommend colonoscopy for stent removal, instead advising patients to wait for the stent to pass naturally. However, if the stent has not passed within 2 weeks, follow-up colonoscopy is performed to ensure its removal.

Appendiceal perforation, occurring either intraoperatively or postoperatively, is regarded as the most severe complication 20 . Notably, no cases of perforation were observed in our study. In contrast, Li et al. reported a case in which a patient with a large fecalith experienced perforation following its removal 21 . This aligns with findings from Mällinen et al., who identified appendiceal fecaliths as an independent risk factor for perforation due to their potential to cause mucosal damage 22 23 . To mitigate this risk, we avoided direct contact between the cholangioscope and fecaliths during the procedure. Instead, fecaliths were gently flushed out using a water stream under direct visualization. Our results suggested that operating under direct visualization may reduce likelihood of appendiceal perforation.

Another noteworthy aspect of our study was assessment of patient satisfaction during follow-up. The majority of patients in the ERAT group expressed high levels of satisfaction, underscoring that this technique was not only effective but also well-received, making it a promising candidate for broader clinical implementation. The patient satisfaction rate following LA was below 80%, largely attributable to visible postoperative surgical scars observed in a subset of cases. In contrast, many patients in the ERAT group did not present with fever or elevated inflammatory markers prior to treatment, suggesting a milder disease state and potentially contributing to more favorable perceptions of the procedure. At our hospital, patients with severe symptoms are typically managed surgically in the Emergency Department, whereas those with milder symptoms are referred to the Gastroenterology Department. Although PSM substantially minimized intergroup differences, residual selection bias could not be entirely eliminated and may have confounded study outcomes.

Safety and efficacy of ERAT in diagnosing and treating appendicitis have been well-documented in several controlled studies 24 25 26 27 . Compared with surgical intervention, ERAT offers the advantages of faster patient recovery and reduced financial burden associated with appendectomy. However, its application is predominantly limited to treatment of acute uncomplicated appendicitis and it is not yet a feasible substitute for surgery in more complex cases. Ding et al. have reported an innovative approach using ERAT to incise abscesses longitudinally or in a cross shape, enabling drainage of pus into the intestinal cavity in 13 patients. Postoperative abdominal ultrasound revealed significant abscess reduction in five cases 24 . This novel technique for managing complex appendicitis highlights the potential for further advancements in ERAT methodologies. We believe that integration of ERAT with the cholangioscope holds substantial promise in driving these innovations.

Current clinical guidelines endorse antibiotic therapy as a viable treatment option for acute uncomplicated appendicitis 14 . A large randomized controlled trial has demonstrated that antibiotic therapy is not inferior to appendectomy in treating acute appendicitis 28 . In our study, nearly all patients received antibiotics, making it difficult to determine whether therapeutic outcomes were primarily due to direct visualization ERAT or the antibiotics themselves. This underscored the need for future observational studies to evaluate outcomes of direct visualization ERAT with and without adjunctive antibiotic therapy.

Nonetheless, this study has certain limitations, including the retrospective design, relatively small sample size, and inability to control for antibiotic use. Follow-up assessments were conducted via telephone, lacking objective measures such as standardized satisfaction questionnaires or clinical examinations. In addition, a relatively short follow-up period that might have overlooked cases of recurrence and potential selection bias favoring patients with milder symptoms for ERAT represent notable constraints. These factors highlighted the need for more robust studies to validate our findings and refine application of ERAT in clinical practice.

## Conclusions

In conclusion, direct visualization ERAT represents a significant advancement in management of acute uncomplicated appendicitis, offering a novel therapeutic option with considerable benefits. This technique has demonstrated comparable efficacy to LA, while offering additional advantages such as appendix preservation, rapid pain relief, and reduced procedure trauma. Despite these merits, its current use remains largely confined to uncomplicated cases of appendicitis. Looking ahead, continued innovation in endoscopic techniques is expected to expand potential applications of ERAT, paving the way for its use in managing more complex clinical scenarios.

## Notes

Correction **Direct visualization endoscopic retrograde appendicitis therapy versus laparoscopic appendectomy for management of acute uncomplicated appendicitis** Jun-yu Pan, Hui-xin Zhi, Jie-li Chen et al. Endoscopy International Open 2025; 13: a26386177. DOI: 10.1055/a-2638-6177 In the above-mentioned article there was an error in a sentence on page E7 (left-hand column, end of first paragraph). The correct wording is: However, if the stent has not passed within 2 weeks, follow-up colonoscopy is performed to ensure its removal. This was corrected in the online version on 19.08.2025.
On 27.10.2025 the author's affiliation and correspondence address were corrected in the online version.
