# Correction: Direct visualization endoscopic retrograde appendicitis therapy versus laparoscopic appendectomy for management of acute uncomplicated appendicitis

**DOI:** 10.1055/a-2684-7813

**Published:** 2025-08-19

**Authors:** Jun-yu Pan, Hui-xin Zhi, Jie-li Chen, Hao-xin Chen, De-feng Li, Jun Yao, Li-sheng Wang

**Affiliations:** 1 Department of Gastroenterology, Shenzhen People’s Hospital, The Second Clinical Medical College, Jinan University, Shenzhen, 518020, China



In the above-mentioned article there was an error in a sentence on page E7 (left-hand column, end of first paragraph). The correct wording is: However, if the stent has not passed within 2 weeks, follow-up colonoscopy is performed to ensure its removal. This was corrected in the online version on 19.08.2025.

On 27.10.2025 the author's affiliation and correspondence address were corrected in the online version.

